# Continued Study of the Relations of the Frontal Sinus to the Antrum

**Published:** 1897-11

**Authors:** Thomas Fillebrown

**Affiliations:** Boston, Mass.


### Continued Study of the Relations of the Frontal Sinus to the Antrum.

BY DR. THOMAS FILLEBROWN, BOSTON, MASS.

Abstract of a paper read at the American Dental Association, Old Point omfort, Va., August, 1897.

Dr. Fillebrown in this paper reviewed briefly his report of last year upon eight instances of what has been considered an abnormal anatomical condition, namely: When the infundibulum continues directly to, and terminates in the foramen of the antrum, a fold of mucous membrane extending above the foramen forming a pocket, from the bottom of which the opening into the antrum is situated, thus directing any discharge coming down the infundibulum into the antrum, no abnormal discharge from the frontal sinus escaping into the nasal passage until the antrum was so filled as to cause a backward overflow. Finding this condition to exist in eight subjects examined by himself seemed to imply that the presence of this pocket membrane was the normal formation.

In the present paper Dr. Fillebrown reports the examination of fifteen heads in the Harvard dissecting room, in every one of which this condition was found to exist (with some minor variations only sufficient to prove the rule), giving a total of twenty-three cases, a number sufficient in his judgment to establish the fact of the normality of the anatomy of the parts.

